# An analysis of the economic impact of smoking cessation in Europe

**DOI:** 10.1186/1471-2458-13-390

**Published:** 2013-04-25

**Authors:** David Cohen, M Fasihul Alam, Paul S Jarvis

**Affiliations:** 1 Health Economics and Policy Research Unit, Faculty of Health, Sport and Science, University of Glamorgan, Pontypridd, Mid Glamorgan CF37 1DL, UK

**Keywords:** Smoking, Modelling, Cost, Policy

## Abstract

**Background:**

There is much evidence that smoking cessation interventions are both clinically and cost effective but these results relate only to the specific study populations involved in the studies. The present study aimed to compare and contrast results obtained when the effects of smoking cessation are modelled for several different European countries.

**Methods:**

Local investigators collected data relating to several smoking related diseases. Costs and disease rates were then modelled up to 2030 for reductions in smoking of 3%, 15% and 30% using an epidemiological modelling tool, PREVENT.

**Results:**

Models could not be constructed for some countries due to lack of data while for others substantial amounts of data had to be imputed. In all cases, disease rates fall when smoking cessation occurs. Overall costs initially fall before eventually rising as lives are saved and the population ages, leading to negative savings in some cases by the end of the modelled period. The speed and magnitude with which these effects occur are diverse for different countries.

**Conclusions:**

Health and economic results for different countries vary significantly for the same reductions in smoking. This suggests that it may be inappropriate to assume that evidence from one country will produce similar health and economic effects if the same levels of smoking cessation were achieved in another country which has evident messages for health policy. Problems with obtaining data also highlight the difficulties associated with modelling such scenarios and underline the need for relevant data to be routinely collected in all countries.

## Background

Evidence based medicine is now an established paradigm within health care [1]. Growing recognition that resources for health care are scarce has led to broad acceptance that the evidence base should include economic as well as clinical evidence. In the UK this is reflected in the work of the National Institute for Health and Clinical Excellence (NICE) whose national guidance on health care for England and Wales is explicitly informed by evidence of cost effectiveness as well as clinical effectiveness [2].

Smoking cessation is one aspect of health policy where the evidence of both clinical and cost effectiveness is strong [3-6]. The empirical studies which provide this evidence, however, reflect the way that smokers responded to smoking cessation interventions in the countries where the studies took place. Clearly, cultural and other differences mean that it cannot be assumed that smokers in other countries will necessarily respond in exactly the same way as they did in the countries of observation. There is thus a potential to misinform if evidence from one country is used to inform policy in another.

Similarly, estimates of the long term health and economic effects which result from reduced smoking are normally derived from mathematical models populated with data from the countries where the modelling exercises took place. These results could also potentially misinform health policy in other countries which may have different rates of smoking prevalence, incidence of smoking related diseases, mortality from those diseases, health service costs, etc.

As a part of a larger project, the present study set out to identify which of 29 European countries participating in the PESCE project (General Practitioners and the Economics of Smoking Cessation in Europe), could provide sufficient data to populate an epidemiological model (PREVENT [7]) which could be used to predict the health and health service cost effects of reduced smoking in those countries.

The aim of the study was to predict the effects of a given reduction in smoking on smoking related disease incidence, mortality and health service costs in each of 29 European countries which could provide sufficient data to allow the PREVENT model to be run for that country and to consider the implications for national policies in the light of differences in results between countries.

This article reports on the findings from the analysis, describing the effects that have been observed and recommendations are made for how future analysis can be improved.

## Methods

### Identifying achievable reductions in smoking

A range of potentially achievable smoking reductions was selected from a recent study commissioned by the UK National Institute for Health and Clinical Excellence [8]. This study reviewed the UK literature on smoking cessation interventions delivered by the National Health Service (NHS), in the workplace and by mass media, and developed a model to assess their cost effectiveness. This approach has an advantage over direct comparisons of cost effectiveness from published studies as the methods employed in the individual evaluations inevitably vary.

Instead, the review team applied a consistent methodology using data extracted from the studies in the review. This involved modelling a hypothetical cohort of 1000 smokers using the costs and cessation effects reported for each intervention together with consistently applied data on mortality by age, gender and smoking status, costs of smoking related diseases and the utilities (health related quality of life) associated with each disease. The resulting cost effectiveness ratios could thus be directly compared as differences would be due solely to differences in costs and effects rather than inconsistent evaluation methods. Table 1 shows the results for the least and most effective smoking cessation interventions included in the NICE exercise together with a mid-range intervention. Although different rates of reduction in smoking might be seen in other countries, these 3 rates (3%, 15% and 35%) were selected for the present exercise only to illustrate low, medium and high effects from smoking cessation interventions. They are of increasing intensity and increasing effectiveness in terms of reductions in smoking as compared with ‘no intervention’ in each case. All demonstrate dominance over ‘no intervention’ meaning that each is both more effective and less costly than doing nothing and hence is unambiguously more cost effective than doing nothing.

**Table 1:** Summary results of cost effectiveness of 3 smoking cessation interventions

**Compared to “no intervention”**	**Effectiveness**	**Duration of intervention**	**Incremental cost**	Incremental QALYs (*)	ICER (**)
Brief Intervention (BA)	3%	3 minutes of GP time	- £12	0.01	Dom (***)
BA + self help material + NRT (****) + specialist clinic	15%	4 mins GP time + self help material + NRT + clinic costs	- £115	0.15	Dom
Nicotine patch + pharmacist + behavioural programme	35%	NRT for 5 weeks + 5 pharmacist consultations + 5 behavioural clinic visits	- £222	0.30	Dom

Source: adapted from [8].

(*) QALY = Quality Adjusted Life Year: a measure capturing both length of life and health state preference adjusted quality of life.

(**) ICER = Incremental Cost Effectiveness Ratio: shows the extra cost of producing one extra QALY for this intervention as compared with no intervention.

(***) Dom = Dominant: A dominant result occurs when the net cost of the intervention is both lower than that of the comparator (in this case no intervention) and also produces more output (QALYs). A dominant result is unambiguously more cost effective.

(****) Nicotine Replacement Therapy.

### The PREVENT model

PREVENT is a Public Health model that links changes in risk factor exposure to changes in risk factor related disease specific outcomes and to changes in generic health outcomes [7]. Despite its venerable age, PREVENT is still being developed. Recently, the central algorithm that relates risk factor change to disease incidence change was modified from an age group perspective to a cohort perspective. This latest version of the PREVENT model was used to estimate reductions in incidence, mortality and health service costs of 4 smoking related diseases (lung cancer, coronary heart disease (CHD), chronic obstructive pulmonary disease (COPD) and stroke) and health service costs.

The model was initially run for the UK where it was known that data were available and of good quality. These data, however, were not always available in the precise format required for the model. Statistical sources were used for data on population, live births, net migration, trends in smoking prevalence, total mortality, disease specific incidence, prevalence and mortality and total health service costs. Published studies were used for disease specific costs for lung cancer [9], COPD [10], CHD and stroke [11] and for relative risks for lung cancer and CHD [12], COPD [13] and stroke [14].

Data for the other European countries were collected using local investigators who were charged with identifying what data were available in that country. A common pro-forma was used by each local investigator to ensure that the same data were provided using common definitions. As this task involved considerable effort and commitment on the part of the local investigators, the quantity and quality of the data provided would inevitably be dependent at least to a degree on the time and effort that each was willing and able to devote to this task.

Where datasets were incomplete, Netherlands data were often used a basis for estimating proportions of data for other countries. The Netherlands was chosen for imputation purposes as the data supplied for that country were of higher quality than others in terms of providing what the PREVENT model requires. For example, while overall birth rate figures could have been obtained from publicly available datasets for countries that did not supply them, the birth rate input also required a breakdown according to different age groups, necessitating imputations that could only be taken from another country where these had been reported.

Additionally, if the incidence of a disease was not known but the prevalence was known, the ratio of incidence to prevalence for the Netherlands was used to estimate the unknown incidence. PREVENT requires these to be broken down by gender and age. Where only totals were provided, the age/gender breakdowns were similarly estimated based on Netherlands data. Where age but not gender breakdowns were supplied, quantities were split equally between male and female. Where only total health service cost figures were supplied, Netherlands rates were used to estimate age and gender breakdowns for total costs and individual disease costs. Netherlands cost data were imputed where total costs figures from individual countries were not provided.

The DISMOD2 model [15] was used to ensure that figures for each dataset were internally consistent. DISMOD2 is a software tool provided by the World Health Organisation that checks the internal consistency of epidemiological estimates of incidence, prevalence, duration, remission and case fatality for diseases. It requires a minimum of three input variables to be supplied. A remission rate of 0 is input to produce estimates for the datasets when fewer than three of the other variables have available data. For some countries, DISMOD2 estimated figures that were previously unknown, while for others, the figures were altered to ensure that internal consistency was valid.

The main study was undertaken prior to 2010 and based on availability of data, the base year in all cases was 2005. Results show the annual predicted reductions for the years 2010, 2020 and 2030 with reduced smoking in 2005, as compared with its predictions for those years without. Figures represent absolute values for each reported year and are not adjusted for population size. Ratios of predicted values in 2030 to those in 2010 show differences in the timing of effects between countries. All cost data were provided in Euros apart from Switzerland and the UK where conversions at 1 franc = €0.62 and £1 = €1.34were used (exchange rates on August 1, 2007). All costs are in 2005 prices.

## Results

Despite the relatively good availability of data for the UK a number of assumptions were still required. These were due in large part to the fact that the UK is made up of 4 countries; England, Wales, Scotland and Northern Ireland. While the model required data for the UK, some data were available only for Great Britain (England, Scotland and Wales), others for “England and Wales” and others still for each country individually. An additional file (‘Additional file 1’) provides a description of the assumptions that were made when these were necessary and indicate that even for a country with relatively good data, estimates from modelling exercises need to be interpreted with a degree of caution due to the large number of assumptions required.

In addition to the UK, sufficient data were provided to run the PREVENT model for 9 other European countries although adjustments and/or imputations were required in all cases. Results for these countries need to be interpreted with caution as some imputations required strong assumptions. ‘Additional file 2’ reports on how the datasets for each country were completed. For all other countries use of the model was judged to be inappropriate as the extent of missing data was excessive.

Results relating to annual reductions in incidence, mortality and costs of the 4 smoking related diseases are summarised in Table 2. All countries show important reductions in health service costs as well as incidence and mortality for the four diseases combined. Orders of magnitude vary considerably as anticipated due inter alia to differences in population size. However, relationships not directly related to population such as that between a country’s long term and short term effects also vary considerably. For example, predicted reductions in disease incidence in 2030 in France and Germany are more than treble those in 2010 while for the UK and Poland they are less than double. Similarly the relationship between each country’s reductions in incidence and savings in health service costs also vary widely. For example, the predicted reduction in incidence in 2030 for Poland is roughly twice that for France (1,971 versus 929 cases) while the cost savings are similar (€43,154 versus €44,981).

**Table 2:** Predicted annual reductions in incidence, mortality and health service savings (€000) of 4 diseases due to reductions of 3%, 15%, 35% in smoking; Base year = 2005

	**3% Reduction in smoking**			**15% Reduction in smoking**			**35% Reduction in smoking**		
	**2010**	**2020**	**2030**	**2010**	**2020**	**2030**	**2010**	**2020**	**2030**
**Netherlands**									
Incidence	342	590	759	1709	2951	3802	3989	6894	8914
Mortality	38	210	329	184	1044	1653	429	2438	3872
Saving (€000)	2069	10147	16342	10344	50754	81884	24138	118458	191712
**Austria**									
Incidence	412	446	497	2066	2241	2497	4839	5276	5896
Mortality	15	71	107	76	354	545	174	829	1282
Saving (€000)	3025	10273	14620	15131	51540	73517	35342	120948	173168
**France**									
Incidence	298	684	929	1494	3412	4649	3484	7931	10868
Mortality	63	415	616	318	2067	3079	738	4800	7199
Saving (€000)	4729	24193	44981	23949	120864	224911	55855	281527	524636
**Germany**									
Incidence	602	1681	2333	3005	8401	11695	7008	19571	27403
Mortality	156	898	1364	770	4488	6835	1791	10455	16013
Saving (€000)	1133	5110	9378	5667	25539	46951	13214	59503	109802
**Ireland**									
Incidence	68	132	186	339	659	943	790	1537	2207
Mortality	9	45	74	39	222	377	89	520	881
Saving(€000)	410	2405	4647	2047	12031	23295	4780	28095	54563
**Poland**									
Incidence	1069	1626	1971	5359	8129	9899	12509	18982	23252
Mortality	122	552	764	609	2769	3835	1419	6468	9011
Saving (€000)	5734	27445	43154	28683	137299	216436	66975	320545	507479
**Portugal**									
Incidence	99	214	297	502	1067	1494	1174	2487	3497
Mortality	10	55	90	50	262	459	117	615	1074
Saving (€000)	597	4592	8960	2995	22931	44852	6981	53367	104799
**Romania**									
Incidence	656	799	848	3282	4005	4238	7666	9364	9926
Mortality	7	40	73	41	198	366	98	462	859
Saving (€000)	779	2546	3876	3894	12821	19395	9090	29941	45350
**Switzerland**									
Incidence	28	56	47	139	276	228	327	639	532
Mortality	2	8	14	9	49	71	23	113	166
Saving (€000)	296	2414	3281	1479	12023	16417	3444	27810	38391
**UK**									
Incidence	1218	1799	2237	6091	9013	11220	14218	21058	26330
Mortality	264	960	1369	1328	4806	6883	3100	11288	16175
Saving (€000)	9865	30532	40528	49280	152744	203185	115008	356837	476219

Reported results relate to annual reductions but these effects are clearly cumulative. By 2030, a 3% reduction in smoking in the UK shows a cumulative reduction of 37,428 cases of the 4 smoking related diseases (taken together), a reduction in deaths from these diseases of 19,260 and a saving in health service costs attributable to these diseases of €603.7 million. Figures for a 35% reduction are 440,648 fewer cases, 227,933 fewer deaths and €7.1 billion saving in health service costs.

The effects of reduced smoking on overall health care costs i.e. accounting for the long term health care costs of an increase in the elderly population are shown in Table 3. All countries show overall cost savings in the short term with variable peaks (shown in bold). By 2030, savings become negative in all countries apart from Romania, Switzerland, Portugal and Austria due to the cost of caring for greater number of older people.

**Table 3:** **Predicted savings in *overall *health service costs from reductions of 3%, 15% and 35% in number of smokers (€000)**

	**2010**	**2015**	**2020**	**2025**	**2030**
**3% Reduction in Smoking**					
Romania	772	1873	2459	3086	**3556**
Switzerland	286	1225	2181	**2804**	2667
Portugal	533	1732	2820	**3359**	2433
Austria	2877	5784	**6741**	6157	4372
Netherlands	1760	**3568**	3161	459	-5876
United Kingdom	8512	**13213**	10788	1636	-14791
Ireland	325	**584**	345	-431	-2060
Poland	4607	**5812**	-6950	-33186	-72598
Germany	**247**	-4535	-16373	-36539	-63056
France	**3350**	-6110	-45580	-124140	-232400
**15% Reduction in Smoking**					
Romania	3863	9368	12303	15445	**17804**
Switzerland	1431	6098	10874	**14026**	13357
Portugal	2664	8649	14081	**16801**	12203
Austria	14393	28996	**33863**	31031	22163
Netherlands	8802	**17835**	15803	2312	-29406
United Kingdom	42566	**66987**	53975	8239	-74105
Ireland	1624	**2919**	1727	-2155	-10327
Poland	23045	**29059**	-34866	-166316	-364111
Germany	**1231**	-22667	-81771	-182576	-315440
France	**16760**	-30010	-226010	-618570	-1161660
**35% Reduction in Smoking**					
Romania	9020	21877	28732	36091	**41638**
Switzerland	3336	14083	25183	**32745**	31270
Portugal	6212	20123	32756	**39207**	28616
Austria	33621	67942	**79638**	73363	52904
Netherlands	20538	**41591**	36859	5487	-68668
United Kingdom	99342	**154264**	126052	19552	-173353
Ireland	3790	**6811**	4027	-5033	-24189
Poland	53818	**67782**	-81882	-389582	-853919
Germany	**2866**	-52828	-190275	-425185	-736324
France	**39070**	-67440	-517330	-1431800	-2707280

Base year = 2005.

## Discussion

The PREVENT model predicts important reductions in smoking related disease incidence and mortality and in the health care costs of treating people with these diseases across all 10 European countries following reductions in smoking of 3%, 15% or 35%. These reductions in smoking are based on UK studies and clearly cessation rates may vary between countries, they illustrate differences in the order of magnitude and in the timing of effects which can have important messages for national health policies.

For a number of reasons, these total identified benefits should be regarded as minima. Firstly, they relate to only 4 diseases and it has long been known that smoking increases the risks of many other diseases (e.g. cataracts), increases other risks (e.g. hip fractures) and inhibits recovery from non-smoking related illness (longer post surgery recovery times). It is the cause of illness in non-smokers who are exposed to second-hand smoke (passive smoking) and has long been known to lead to higher levels of low birth rate babies in women who smoke when pregnant. (See [16] for summary of effects of smoking).

Reduced smoking can also lead to non-health benefits particularly in terms of productivity gains to the economy. Workers who smoke have higher rates of sickness absence from work than do non-smokers [17] which was estimated to be responsible for 50 million lost working days per year in the UK [18]. In Scotland alone, the total annual costs due to such additional sickness absence from work by smokers has been estimated at £40 million (€47.2 million) [19]. In addition there are other benefits of reduced smoking such as fewer fires. It has been estimated that 10% of all fires in the UK are due to cigarettes and a further 9% to use of matches [20].

Reductions in smoking related mortality, however, mean more people living to old age which has implications for long term health care costs. The model predicts initial overall health care savings in all countries which reach a peak and then decline, becoming negative in six of the ten modelled countries by the end of the modelled period. Savings become negative when the population structure contains a higher proportion of older people, which will eventually occur in all ten cases but takes more time in countries that start with a relatively young population. Negative overall health care savings however, cannot be interpreted as ‘negative’ results since they are the direct result of people living longer, healthier lives which is the explicit objective of smoking cessation policies – as it is for all health care interventions. The decision to treat a patient suffering a myocardial infarction (MI) is unlikely to include consideration of the fact that saving his life means he will live to old age and become a burden on the health service. There is no reason why future health service costs should have any more influence on the decision to prevent the MI in the first place.

The fact that sufficient data to run the PREVENT model were obtained from only 10 of 29 European countries does not mean that data for the remaining 19 do not exist nor that the data provided for these 10 are necessarily as complete as might have been possible. Reliance on local researchers in this many countries meant that some variation in terms of the effort and rigor applied to obtaining data was inevitable. Despite this caveat, results demonstrate that the quantity and quality of data available for purposes of predictive modelling can vary significantly across European countries. Nevertheless, deficiencies in the datasets were clearly often due to those data not being collected and improvements in routine collection of data such as Burden of Disease data and performing Costs of Illness studies would greatly assist future smoking cessation research in Europe.

Differences in the predicted impact of reduced smoking vary considerably between countries which has important implications for evidence based policy. For example, while the population of Germany is more than double that of Poland (82.5 million versus 38.1 million) reductions in disease incidence following a 3% reduction in smoking in 2005 are considerably greater for Poland in the short term (1069 versus 602 cases avoided in 2010) but in the long term the situation is reversed (1,971 versus 2,333 cases avoided in 2030). The overall health service cost saving for both countries, however, peak and become negative fairly quickly (Germany, peak in 2010 negative in 2015, Poland peak in 2015 negative in 2020) as compared with say Romania where the savings are still rising in 2030 and, due to that being the last year modelled, will possibly continue to rise even beyond that.

This study has demonstrated that there are dangers in using evidence produced in one European country to inform smoking policy in another. Even neighbouring countries that may superficially appear to be similar in terms of some demographics have shown large disparities in the outputs generated in this study, demonstrating the need for careful analysis of accurate and complete local datasets with a particular emphasis upon collecting burden of disease and cost of illness data.

### Limitations of the study

Considerable care needs to be taken in interpreting these results which should be seen as illustrative. The completeness of data to meet the requirements of the PREVENT model varied considerably between countries with fairly heroic assumptions being required in some cases as shown in ‘Additional file 2’. Apart from the UK, datasets for all countries required some adjustment or imputation using data from another country. These varied from minimal, for example in the case of the Netherlands where all that was required was to apply age breakdowns from France to Netherlands birth rates, to severe, for example in the case of Austria, Portugal and Poland which did not provide any cost figures and where Netherlands costs were imputed. Clearly the accuracy of any prediction varies with the number and severity of adjustments and imputations required. The purpose of the study, however, was to illustrate how the effects of reduced smoking can differ between countries.

Results are reported as absolute values rather than as rates. Clearly the implications of any given reduction in the absolute number of new cases, deaths or health care costs have to be interpreted locally with regard to the size of the population. Converting values into rates, however, requires predicting population growth rates in addition to the predicting changes in the variables examined here. This would not have affected the main messages from the study.

## Conclusions

In all countries modelled, healthcare costs initially fall before eventually rising as the population ages, however the speed at which these changes occur varies greatly between different countries. All countries show initial reductions in health service costs as well as incidence and mortality for the diseases combined, however the magnitude of the figures varies considerably between different countries, even for countries of similar sized populations. Lack of data has hindered the precision of results obtained and suitable data should be routinely collected locally in order to accurately model the effects of smoking cessation. With more accurate data, the results which have been obtained through the analysis in this study can be interpreted with greater confidence and allow policy makers to make informed choices about the costs and benefits of the implementation of smoking cessation programmes. It is clear from this analysis that outcomes will vary according to local factors and that it is not appropriate to assume that uniform changes in smoking patterns will lead to identical effects in different countries.

## Abbreviations

CHD: Coronary Heart Disease; COPD: Chronic Obstructive Pulmonary Disease; NICE: National Institute for Health and Clinical Excellence; NHS: National Health Service; PESCE: General Practitioners and the Economics of Smoking Cessation in Europe.

## Competing interests

The authors declare that they have no competing interests.

## Authors’ contributions

DC led the economic element of the PESCE project, designing the study and drafted the manuscript. MFA participated in design of the study and the analysis of UK data and helped to draft the manuscript. PSJ analysed other European data and helped to draft the manuscript. All authors read and approved the final manuscript.

## Authors’ information

DC is Director, Health Economics and Policy Research Unit (HEPRU) at the University of Glamorgan. MFA and PSJ are Senior Research Fellow and Research Fellow respectively within HEPRU.

## Pre-publication history

The pre-publication history for this paper can be accessed here:

http://www.biomedcentral.com/1471-2458/13/390/prepub

## Supplementary Material

Additional file 1 Sources of data used and assumptions: UK dataset. Click here for file

Additional file 2 Adjustments and imputations to European country datasets. Click here for file

## References

[B1] Evidence Based Medicine Working Group Evidence based medicine: a new approach to teaching the practice of medicine J Am Med Assoc 1992 268 242 245 10.1001/jama.1992.03490170092032 1404801

[B2] National Institute for Clinical Excellence Guide to the methods of technology appraisal 2004 London: National Institute for Clinical Excellence 27905712

[B3] Song F Raftery J Aveyard P Hyde C Barton P Woolacott N Cost-effectiveness of pharmacological interventions for smoking cessation: a literature review and a decision analytic analysis Medical Decision Making 2002 22 Supplement S26 S37 12369228 10.1177/027298902237708

[B4] Feenstra TL Hamberg-van Reenen HH Hoogenveen RT Rutten-van Molken MP Cost-effectiveness of face-to-face smoking cessation interventions: a dynamic modeling study Value Health 2005 8 3 178 190 10.1111/j.1524-4733.2005.04008.x 15877590

[B5] Godfrey C Parrott S Coleman T Pound E The cost-effectiveness of the English smoking treatment services: evidence from practice Addiction 2005 100 Suppl 2 70 83 15844290 10.1111/j.1360-0443.2005.01071.x

[B6] Stapleton J Cost effectiveness of NHS smoking cessation services [http://www.ash.org.uk/files/documents/ASH_427.pdf] Accessed May 2, 2012

[B7] Gunning-Schepers L The health benefits of prevention: a simulation approach Health Policy 1989 12 1–2 1 255 10303654

[B8] National Institute for Health and Clinical Excellence Cost-Effectiveness of Interventions for Smoking Cessation [http://www.nice.org.uk/nicemedia/pdf/WorkplaceInterventionsPromoteSmokingCessationEconomicReport1.pdf] Accessed May 2, 2012

[B9] Wolstenholme J Whynes D The hospital cost of treating lung cancer in the United Kingdom Br J Cancer 1999 80 215 218 10.1038/sj.bjc.6690341 10389998 PMC2363001

[B10] Britton M The burden of COPD in the UK: results from the confronting COPD survey Respir Med 2003 97 Sup C S71 S79 12647945 10.1016/s0954-6111(03)80027-6

[B11] Luengo-Fernandez R Leal J Gray A Petersen S Rayner M Cost of Cardiovascular disease in the United Kingdom Heart 2006 92 1384 1389 10.1136/hrt.2005.072173 16702172 PMC1861058

[B12] Select Committee on Health Minutes of Evidence. House of Commons 2000 London: The Stationary Office

[B13] Wilson D Adams R Appleton S Ruffin R Difficulties identifying and targeting COPD and population-attributable risk of smoking for COPD Chest 2005 128 2035 2042 10.1378/chest.128.4.2035 16236852

[B14] Shinton R Beevers G Meta-analysis of relation between cigarette smoking and stroke Br Med J 1989 298 789 794 10.1136/bmj.298.6676.789 2496858 PMC1836102

[B15] World Health Organisation[http://www.who.int/healthinfo/global_burden_disease/tools_software/en/index.html] Accessed May 2, 2012

[B16] Cohen D Barton G The costs to society of smoking cessation Thorax 1998 53 S2 S38 S42 10193346 10.1136/thx.53.2008.s38 PMC1765898

[B17] Bertera RL The effects of behavioural risks on absenteeism and health care costs in the workplace J Occup Med 1991 33 119 124 10.1097/00043764-199111000-00006 1765851

[B18] Townsend J Crofton J, Wood M Cost effectiveness and mass media programmes of smoking control Smoking control: strategies and evaluation in community and mass media programmes 1986 London: Health Education Council, & Belfast: The Ulster Cancer Foundation, & Edinburgh: Scottish Health Education Group

[B19] Parrott S Godfrey C Raw M Cost of employee smoking in the workplace in Scotland Tob Control 2000 9 187 192 10.1136/tc.9.2.187 10841855 PMC1748323

[B20] Nelson H The Economic Consequences of Smoking in Northern Ireland 1986 Belfast: Ulster Cancer Foundation

