# Supplementary material for: An analysis of the economic impact of smoking cessation in Europe
Source: BMC Public Health. 2013 Apr 25;13:390. doi: 10.1186/1471-2458-13-390 (PMC3644224; doi:10.1186/1471-2458-13-390)
Supplement: Additional file 1 — Sources of data used and assumptions: UK dataset. [file 1471-2458-13-390-S1.doc]

## Sources of data used and assumptions: UK dataset

UK = England, Scotland, Wales and Northern Ireland

GB = England, Scotland and Wales

E&W = England and Wales

NI = Northern Ireland

GROS = General Register Office for Scotland

NISRA = Northern Ireland Statistics and Research Agency

ONS = Office for National Statistics

WCISU = Welsh Cancer Intelligence and Surveillance Unit

HES = Hospital Episode Statistics (England)

HSW = Health Solutions Wales

ISD = Information Services Division (Scotland)

GHS = General Household Survey

BHF = British Heart Foundation

| **Variable** | **Source** | **Assumption** | **Comment** |
| --- | --- | --- | --- |
| **Population** | 1.E&W: (ONS)  2. Scotland: (GROS),  3. Northern Ireland: (NISRA) |  |  |
| **Live births** | 1. ONS 2004  2. GROS 2005 | E&W: live birth data by age of mother and sex of baby for 2004 applied to 2005 | For E&W latest available data by age/sex = 2004 and for Scotland =2005. Added to estimate UK 2005 |
| **Net migration** | ONS 2005 |  | 5 year average (2001-2005) net migration used for UK |
| **Total mortality** | 1. ONS 2005  2. GROS 2005 |  | Total mortality data by age & sex were available for 2005. E&W and Scotland data used as estimate for the UK |
| **Incidence of Lung Cancer (LC)**  ICD10 C33-C34 | 1. England: ONS 2004  2. Wales: WCISU 2005  3. Scotland: ISD 2004 | For England and Scotland, LC incidence data available for 2004. Assumed LC incidence is same for 2005. | LC incidence data available separately England, Wales and Scotland for different calendar years. Added to derive UK |
| **Incidence of COPD**  ICD-10 J44 | 1. England: HES - Primary diagnosis 2004/5  Incidence = hospital admission for first episode. Includes patients admitted in previous years  2. Wales: HSW - Number of admissions, 2004 | Number of admissions by COPD patients in E&W as estimate of COPD incidence in the UK | At the time of data collection, COPD incidence data were available for E&W only |
| **Incidence of CHD** | 1. England: HES  Primary diagnosis 2004-5  2. Wales: HSW No. of admissions, 2004 | E&W data used to estimate CHD incidence for UK | Latest published CHD incidence data for Scotland available only for 1999. Considered this too old to match with E&W data which was available for 2004/5. |
| **Incidence of Stroke** | 1. England: ONS, 1999  2. Scotland: ISD, 1999 | Since there were no published stroke incidence data after 1999, this was used as estimate for 2005 | Stroke incidence data for E&W available from ONS for 1999. Similar data were available for Scotland in 1999. These were added to estimate UK for 2005 |
| **Prevalence of Lung Cancer** | Data for 1999 from [1] | Since no published LC prevalence data by age and sex after 1992, we used this data as an estimate of the base year 2005 | LC estimates from the study are for the UK. |
| **Prevalence of COPD** | GB: ONS GHS 2005  (Table 7.15: Chronic sickness: rate/000 reporting selected longstanding conditions, by sex and age) | Bronchitis and emphysema entries from the table used as estimates for COPD prevalence |  |
| **Prevalence of CHD** | GB: ONS GHS 2003 | Prevalence data for 2003 used to estimates 2005 |  |
| **Prevalence of Stroke** | GB: ONS GHS, 2005  (Table 7.15: Chronic sickness: rate/000 reporting selected longstanding conditions, by sex and age) |  |  |
| **Mortality from Lung Cancer** | UK: BHF 2005 (based on the ONS Death Registered by cause for E&W, GRO for Scotland and NI) | Mortality data based on 2004 death registration. Assumed disease specific mortality in 2004 same in 2005 for the UK. |  |
| **Mortality from COPD** | UK: Deaths from different causes 2004.The Burden of Lung Disease. 2nd edition. Published report for the Thoracic Society 2006 (Table 1.1 Deaths from different causes, UK 2004) | Mortality data based on 2004 death registration. Assume disease specific mortality in 2004 will be same in 2005 for UK |  |
| **Mortality from CHD** | UK: BHF 2005 (based on the Death Registered by cause for England and Wales, GRO for Scotland and NI) | Mortality data based on 2004 death registration. Assume disease specific mortality in 2004 will be same in 2005 for UK. |  |
| **Mortality from Stroke** | UK: BHF 2005 (based on the ONS Death Registered by cause for E&W, GRO for Scotland and NI Ireland) | Data based on 2004 death registration. Assume disease specific mortality in 2004 same in 2005 for UK. |  |
| **Health Care Costs Lung Cancer** | GP consultations from [2].  Cost of consultation based on 2005 rate.  Hospital costs from [3] | a) Per patient cost = GP consultation cost + hospitalisation cost for treating a LC patient.  b) health care costs from study are not by age and sex. Apportionment to age/sex by proportions in the Netherlands (Personal communication) |  |
| **Health Care Cost COPD** | From [4] | Health care costs not reported by age and sex. Apportionment method uses as for lung cancer |  |
| **Health Care Cost CHD** | From [5] | Health care costs not reported by age and sex. Apportionment method used as for lung cancer | Estimates based on major study of all UK CVD patients in 2004 |
| **Health Care Costs Stroke** | From [5] | Health care costs not reported by age and sex. Apportionment method used as for lung cancer | As above |
| **Total Health Care Costs** | House of Commons Health Committee report, 2006. Per capita costs from hospital and community health services expenditure by age, UK 2003/2004. | Total cost data not available by sex, so assumed vary by age but equal for male/female.  2005 UK population used to convert total health care cost to per capita costs |  |
| **Smoking prevalence** | UK: GHS | Data only for alternate years. Missing years imputed using average for years before and after. |  |
| **Relative Risk LC** | Minutes of Evidence, Select Committee on Health, House of Commons, The UK Parliament, Prepared in 2000 |  | From [6] |
| **Relative Risk COPD** | From [7] |  | This Australian study was used in the absence of a proper UK study reporting RR of COPD |
| **Relative Risk CHD** | Minutes of Evidence, Select Committee on Health, House of Commons, The UK Parliament, Prepared in 2000 |  |  |
| **Relative Risk Stroke** | From [8] |  |  |
