# Supplementary material for: An analysis of the economic impact of smoking cessation in Europe
Source: BMC Public Health. 2013 Apr 25;13:390. doi: 10.1186/1471-2458-13-390 (PMC3644224; doi:10.1186/1471-2458-13-390)
Supplement: Additional file 2 — Adjustments and imputations to European country datasets. [file 1471-2458-13-390-S2.doc]

## Adjustments and imputations to European country datasets

Austria:

Birth rate age categories were imputed from France data.  Mortality data for all diseases were supplied. Incidence figures for stroke were supplied as were total incidence of lung cancer, but age breakdowns for lung cancer and all figures for COPD and CHD were imputed using Netherlands proportions of mortality data. DISMOD2 was then used to provide prevalence figures as well as ensure the figures were internally consistent. As no cost data were provided, Netherlands data were copied for total cost and disease costs.  Smoking prevalence data were supplied only for 1995.  This figure was imputed for 1975-2005.

Ireland:

Mortality data were supplied for all diseases, so prevalence and incidence of all other diseases were imputed from Netherlands rates, with the exception of lung cancer incidence which were also supplied. DISMOD2 was then applied to ensure internal consistency. An overall cost figure was supplied, so Netherlands rates were used to estimate age and gender breakdowns for total costs and individual disease costs. Smoking prevalence data were supplied for the 3 most recent years. These were split equally for each gender and the earliest year’s figures imputed for the previous 30 years.

Portugal:

Birth rate gender breakdowns were estimated by dividing totals equally. DISMOD2 was used to ensure internal consistency and also estimate missing values for incidence rates for COPD and prevalence values for lung cancer, CHD and stroke. All cost data were copied from Netherlands figures. Smoking data were supplied for one year, which was copied for all missing years.

Poland:

Mortality data were supplied for all four diseases, while incidence figures were imputed using Netherlands rates. DISMOD2 was then used to estimate all prevalence figures and ensure internal consistency. As no cost figures were supplied, these were copied from Netherlands figures. Smoking data were supplied for seven time points dating back 31 years. Figures were repeated where necessary to fill in gaps and cover the entire time period.

Romania:

Migration age breakdowns were supplied, but gender breakdowns were estimated by dividing figures equally. Birth rate age and gender breakdowns were imputed using rates from Poland.  Incidence values were available for all diseases (gender breakdowns were estimated for Stroke, CHD and COPD). Mortality totals were supplied but age and gender breakdowns were imputed from Netherlands proportions. All prevalence values were estimated by DISMOD, which also ensured internal consistency. For total costs and disease specific costs, overall totals were supplied with age and gender breakdowns estimated using Netherlands proportions. Smoking prevalence values were available for 2 time points and imputed to cover the entire period.

Germany:

Birth rate figures were not supplied for all age categories. These were imputed from French figures. Incidence and prevalence figures for COPD were imputed using Netherlands rates. DISMOD2 was used to ensure incidence, prevalence and mortality figures were internally consistent for all diseases. Smoking prevalence data were supplied for 5 separate time points, with figures repeated to provide totals for missing years.

Netherlands:

Birth rate age breakdowns were imputed using French proportions. DISMOD2 was used to ensure incidence, prevalence and mortality figures were internally consistent.

France:

Migration age breakdowns were imputed using Netherlands proportions. Age breakdowns for disease costs and total costs were also imputed from Netherlands proportions. DISMOD2 was used to estimate prevalence figures for all diseases and to ensure all incidence, prevalence and mortality figures were internally consistent. Smoking prevalence data were supplied only for 2004 but were imputed for missing years.

Switzerland:

Migration age breakdowns were imputed using Netherlands proportions. DISMOD2 was used to estimate, incidence figures for lung cancer and stroke, and prevalence figures for CHD and COPD. DISMOD2 also ensured incidence, prevalence and mortality figures for all diseases were internally consistent. Disease costs were imputed by using Netherlands proportions of total costs. Smoking prevalence figures go back to 2001, therefore these figures were repeated to provide figures for previous missing years.
